# Supplementary material for: Household Income Is Associated with Chronic Pain and High-Impact Chronic Pain among Cancer Survivors: A Cross-Sectional Study Using NHIS Data
Source: Cancers (Basel). 2024 Aug 15;16(16):2847. doi: 10.3390/cancers16162847 (PMC11353052; doi:10.3390/cancers16162847)
Supplement: Supplementary file 1 [file cancers-16-02847-s001.zip › cancers-3134893-supplementary.pdf]

Supplementary materials

Table S1: Frequency of missing variables.

	Missing n	weighted %						
	Overall N=4,585		<200% FPL n=1,673		200%-399% FPL n=1885		≥400% FPL n=2,846	
**Age (years)**	4	0.12	0		2	0.1	3	0.1
**Marital status**	52	1.20	27	1.9	21	1.1	17	0.6
**Education**	27	0.58	16	1.1	4	0.2	10	0.3
**Insurance covered**	4	0.10	1	0.1	2	0.1	1	0.0
**BMI**	104	2.40	30	1.9	53	2.7	56	2.2
**Smoking history**	38	0.88	22	1.6	15	0.7	10	0.4
**Comorbidities**								
Diabetes (Yes)	3	0.04	0		3	0.03	0	
Hypertension (Yes)	3	0.04	2	0.02	2	0.02	0	
Arthritis (Yes)	3	0.05	4	0.2	1	0.02	1	0.04
Depression (Yes)	6	0.08	3	0.02	3	0.04	1	0.01
Anxiety (Yes)	7	0.12	0		6	0.07	2	0.04

Abrrevations: FPL -Federal Poverty Level

Table S2: Prevalence of high-impact pain-relieving methods adopted by cancer survivors by Federal Poverty Level (FPL).

**High impact pain**<br>Unweighted n, weighted %	Overall<br>1184	<200% FPL<br>476 (%)	200%-399% FPL<br>338 (%)	≥400% FPL<br>370 (%)
Opioid use				
Opioid use in the past 12 months				
Yes	449 (37.9)	210 (43.0)	111 (33.7)	128 (35.2)
No	674 (56.6)	245 (52.3)	210 (60.5)	219 (58.5)
Not asked/refused/missing	61 (5.5)	21 (4.7)	17 (5.7)	23 (6.2)
Opioid use in the past 3 months				
Yes	345 (29.0)	166 (34.1)	86 (26.8)	93 (27.8)
No	104 (8.8)	44 (8.9)	25 (6.9)	35 (10.5)
Not asked/refused/missing	735 (62.1)	266 (60.0)	227 (66.3)	242 (64.2)
Acute opioid use in the past 3 months				
Yes	205 (16.7)	100 (18.7)	52 (16.3)	53 (14.5)
No	140 (12.3)	66 (15.4)	34 (10.4)	40 (10.2)
Not asked/refused/missing	839 (71.0)	310 (65.9)	252 (73.2)	277 (75.2)
Non-opioid methods				
Physical therapy				
Yes	260 (20.4)	91 (15.9)	68 (17.7)	101 (28.5)
No	923 (79.5)	384 (4.0)	270 (82.3)	269 (71.5)
Not asked/refused/missing	1 (0.1)	1 (0.1)	0	0
Chiropractic care				
Yes	118 (9.2)	35 (6.1)	40 (11.5)	43 (11.1)
No	1062 (90.4)	438 (93.1)	298 (88.5)	326 (88.7)
Not asked/refused/missing	4 (0.4)	0.3 (0.8)	0	0.1 (0.2)
Talk, Cognitive-behavioral therapy				
Yes	36 (2.3)	15 (2.0)	9 (2.1)	12 (2.8)
No	1144 (97.4)	458 (97.4)	328 (97.7)	358 (97.2)
Not asked/refused/missing	4 (0.3)	3 (0.6)	1 (23.6)	0
Yoga, Tai Chi, or Qi Gong				
Yes	88 (7.2)	31 (8.3)	24 (5.6)	33 (7.2)
No	1094 (92.6)	443 (91.2)	314 (94.4)	337 (92.8)
Not asked/refused/missing	2 (0.2)	2 (0.5)	0	0
Massage				
Yes	182 (14.2)	49 (8.9)	63 (16.8)	70 (18.5)
No	999 (85.5)	424 (90.5)	275 (83.2)	300 (81.5)
Not asked/refused/missing	3 (0.2)	3 (0.6)	0	00
Meditation				
Yes	209 (15.6)	86 (17.9)	64 (15.5)	59 (12.9)
No	972 (84.2)	387 (81.4)	274 (84.5)	311 (87.1)
Not asked/refused/missing	3 (0.3)	3 (0.7)	0	0
Other				
Yes	445 (35.0)	167 (31.4)	119 (35.6)	159 (39.0)
No	736 (64.8)	206 (68.0)	219 (64.4)	211 (61.0)
Not asked/refused/missing	3 (0.2)	3 (0.6)	0	0

Table S3: Multivariable logistic regression model for chronic pain and Federal Poverty Level (FPL) using the National Health Interview Survey (NHIS), 2019-2020.

**Variables**	**Chronic pain (n=4,585)**			**High-impact pain (n=1,184)**		
	aOR	95% CI	*P-value*	aOR	95% CI	*P-value*
Federal Poverty Level			.0007			.073
<200%	1.60	1.25 – 2.05		1.71	1.06 – 2.75	
200%-399%	1.18	0.96 – 1.46		1.16	0.76 – 1.80	
≥400%	**Ref**	--		**Ref**	--	
Age (years)			.0007			.19
18-44	**Ref**	--		**Ref**	--	
45-64	1.63	1.04 – 2.57		1.68	0.77 – 3.65	
≥65	1.11	0.70 – 1.75		1.27	0.57 – 2.82	
Sex			.30			.66
Female	**Ref**	--		**Ref**	--	
Male	1.11	0.91 – 1.34		1.08	0.74 – 1.60	
Race/ethnicity			.91			.33
NH-White	**Ref**	--		**Ref**	--	
NH-Black	0.98	0.70 – 1.38		1.34	0.75 – 2.38	
NH-Asian	1.11	0.51 – 2.44		1.56	0.58 – 2.87	
Hispanic	1.08	0.74 – 1.57		1.54	0.75 – 3.16	
Other	1.27	0.74 – 2.20		1.21	0.49 – 2.99	
Marital status			.61			.21
Not married	0.95	0.78 – 1.16		1.26	0.88 – 1.81	
Married/Living with partner	**Ref**	--		**Ref**	--	
Education			.69			.21
Less than high school	1.15	0.82 – 1.61		1.57	0.94 – 2.63	
High-school or GED	1.04	0.84 – 1.29		1.20	0.82 – 1.75	
Some college or more	**Ref**	--		**Ref**	--	
Insurance			.40			.32
Covered	1.40	0.80 – 2.47		1.86	0.77 – 4.49	
Not covered	**Ref**	--		**Ref**	--	
BMI			.0002			.36
Normal weight	**Ref**	--		**Ref**	--	
Underweight	1.10	0.54 – 2.23		1.55	0.53 – 4.55	
Overweight	0.96	0.79 – 1.18		0.86	0.56 – 1.31	
Obese	1.52	1.22 – 1.89		1.21	0.81 – 1.82	
Urbanicity			.41			.12
Rural	**Ref**	--		**Ref**	--	
Urban	0.90	0.70 – 1.15		1.41	0.91 – 2.19	
Diabetes			.03			.98
No	**Ref**	--		**Ref**	--	
Yes	1.29	1.02 – 1.65		0.99	0.65 – 1.53	
Hypertension			.02			.03
No	**Ref**	--		**Ref**	--	
Yes	0.80	0.65 – 0.97		0.69	0.49 – 0.96	
Arthritis			<.0001			.80
No	**Ref**	--		**Ref**	--	
Yes	4.02	3.33 – 4.84		0.95	0.66 – 1.39	
Depression			.0001			.45
No	**Ref**	--		**Ref**	--	
Yes	1.65	1.28 – 2.13		0.86	0.58 – 1.27	
Anxiety			.02			.0001
No	**Ref**	--		**Ref**	--	
Yes	1.38	1.06 – 1.82		2.29	1.51 – 3.48	

Abbreviations: aOR – adjusted odds ratio, CI – confidence interval, BMI – body mass index, GED – General Education Diploma, NH – non-Hispanic.
